# Functional metagenomic analysis of dust-associated microbiomes above the Red Sea

**DOI:** 10.1038/s41598-019-50194-0

**Published:** 2019-09-24

**Authors:** Nojood A. Aalismail, David K. Ngugi, Rubén Díaz-Rúa, Intikhab Alam, Michael Cusack, Carlos M. Duarte

**Affiliations:** 1 Red Sea Research Centre (RSRC) and Computational Bioscience Research Center (CBRC), King Abdullah University of Science and Technology (KAUST), Thuwal, 23955 Saudi Arabia; 2 Department of Microorganisms, Leibniz Institute DSMZ - German Collection of Microorganisms and Cell Culture, Inhoffenstrasse 7B, B38124 Braunschweig, Germany; 3 Computational Bioscience Research Center, King Abdullah University of Science and Technology, Thuwal, 23955 Saudi Arabia

**Keywords:** Microbial ecology, Air microbiology, Metagenomics

## Abstract

Atmospheric transport is a major vector for the long-range transport of microbial communities, maintaining connectivity among them and delivering functionally important microbes, such as pathogens. Though the taxonomic diversity of aeolian microorganisms is well characterized, the genomic functional traits underpinning their survival during atmospheric transport are poorly characterized. Here we use functional metagenomics of dust samples collected on the Global Dust Belt to initiate a Gene Catalogue of Aeolian Microbiome (GCAM) and explore microbial genetic traits enabling a successful aeolian lifestyle in Aeolian microbial communities. The GCAM reported here, derived from ten aeolian microbial metagenomes, includes a total of 2,370,956 non-redundant coding DNA sequences, corresponding to a yield of ~31 × 10^6^ predicted genes per Tera base-pair of DNA sequenced for the aeolian samples sequenced. Two-thirds of the cataloged genes were assigned to bacteria, followed by eukaryotes (5.4%), archaea (1.1%), and viruses (0.69%). Genes encoding proteins involved in repairing UV-induced DNA damage and aerosolization of cells were ubiquitous across samples, and appear as fundamental requirements for the aeolian lifestyle, while genes coding for other important functions supporting the aeolian lifestyle (chemotaxis, aerotaxis, germination, thermal resistance, sporulation, and biofilm formation) varied among the communities sampled.

## Introduction

**Figure 1:**
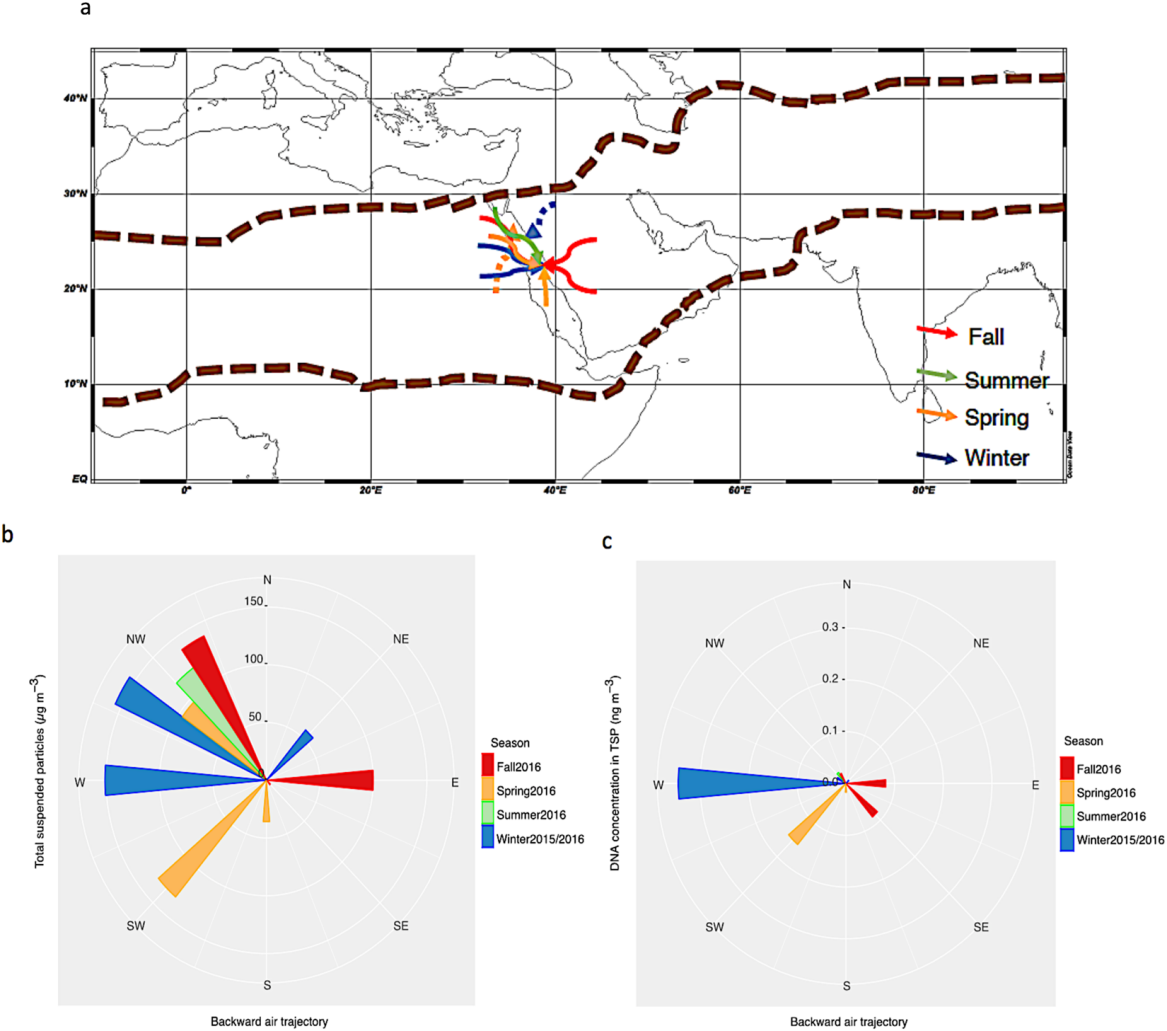
Total suspended particles (TSP) sampling locations and backward air trajectories. **(a)** Map of the Global Dust Belt area within the two brown dashed lines. Solid arrows indicate the land-sea interface air sampling location of eight aeolian samples, dotted arrows indicate the offshore air sampling location of two aeolian samples. Colors of the arrows show the sampling season. (**b)** Polar plot of the TSP concentrations in ten aeolian samples showing their different air backward trajectories, bar colors indicate the sampling season. (**c**) Polar plot of the DNA concentrations in ten aeolian samples showing their different air backward trajectories, bar colors indicate the sampling season.

Desert dust is one of the main sources of aerosols^1^, including both mineral particles and aeolian microorganisms, supplying the atmosphere with a heavy load of microbes that can be transported across large distances^2^. Atmospheric dust loads are concentrated in a broad region extending from the west coast of North Africa, through the Middle East, into Central Asia (approximately 10 to 30°N), deemed the “Global Dust Belt”^3^ (Fig. 1a). These regions represent the main sources of dust to the atmosphere and disproportionately contribute, therefore, to transferring biological particles to the Aeolian microbiome. The Aeolian microbiome, referring to the community of microbes present in the atmosphere, arguably represents the least studied compartment of the microbiome of the biosphere, and yet, it plays a fundamental role in the air chemistry specially in the polluted areas^4^ and as already hypothesized by L. Pasteur, human health, as microorganisms transported through the air may affect the general human and ecosystem health^5^. Hence, most research effort on the Aeolian microbiome has focused on aeolian dust collected in indoor, urban and terrestrial environments^6^, with far less attention devoted to the Aeolian microbiome over the ocean^7^. Whereas the metagenomes analyzed were collected either offshore or at the shoreline (KAUST pier), the back-trajectories of the air masses sampled (Fig. 1) transited over spans of sea (Red Sea, Mediterranean, and Arabian Gulf and Indian Ocean) as well as spans of land. Hence, they likely reflect contributions of microbes aerosolized from land and ocean. Further studies in samples collected offshore in the open ocean, far away from land sources, are required to better characterized the microbiome of the oceanic atmospheric boundary layer, which has been reported to be dominated by microorganisms originated on land^7^. Microorganisms are ejected into the atmosphere through turbulent movement of air over objects and surfaces, a process known as aerosolization. Once in the air, they may remain suspended for longer than a week before precipitation onto surfaces, although calculated median suspension times are only a few days^8^. Strong winds play an important role in transporting aeolian microorganisms far away from the source by the adhesion of microbes to dust particles^9^.

Recent analyses of the aeolian microbiota in the Global Dust Belt paid huge attention to aerosols fungi and prokaryotes, often abundant in airborne microbial communities, which might be harmful to the receiving ecosystems and human populations, such as those in Sahara desert and China^10,11^. These analyses concluded that microbes in the air at these locations originated from North Africa and Asia^12^, which represent the edges of the Global Dust Belt.

However, current understanding of the Aeolian microbiome focusses on quantifying loads and fluxes and characterizing the identity of the microorganisms transported^7,9^. However, the ability of microorganisms to survive in the harsh atmospheric environment (e.g. high UV radiation and heat) is poorly understood, but must be controlled by a set of inherent functions conferring resistance to transport in a dry air medium or particulate Aeolian dust. We provide here the first attempt at describing the Red Sea Aeolian microbiome from a functional metagenomics perspective, and reviewed the literature on the functions postulated to be important in supporting successful Aeolian microbial transport to propose a set of targeted genes that help define the Aeolian lifestyle. As a consequence, a set of functions was selected to be analyzed in the airborne metagenomes, these functions are aerosolization^13^, allowing microbes to be entrained from surfaces (soils, plants or water) to the air facilitated by gas vesicles inside the cells, aerotaxis^14^ and chemotaxis^15^, which are the movement of microbes under the influence of oxygen or chemical gradient that allow them to be positioned at the surface of their habitat, UV radiation^16^ and heat resistance^17^, which function in repairing the DNA damages induced by UV radiation and thermal stresses, germination^18^ and sporulation^19^, where the cells can form spores, which allow them to survive desiccation and exposure to UV radiation and germinate under harsh conditions, and biofilm formation^20^ that enable the microorganisms to attach to surfaces such as dust particles. We propose here this set of functions as a parsimonious set of traits that delineates capacities that, in combination, allow biological particles to survive atmospheric transport.

Metagenomic approaches describing the functional properties and diversity of microbiomes were first introduced by the Global Ocean Expedition, where massive shotgun sequencing was applied to describe microbial and gene diversity in plankton communities in the Sargasso Sea^21^. The quest to characterize microbial life and gene function and diversity in the ocean has continued through global expeditions, such as TARA Oceans^22^ and the Malaspina Circumnavigation Expedition^23^ and global assessments of soil microbiota^24^. However, this powerful approach (whole genome shotgun sequencing) has been applied rarely, to the best of our knowledge, to describe aeolian microbiota, using samples of aeolian microorganisms indoor^25^ and outdoor built urban^26^ environments, compared to hundreds of (WGS) studies published on land and oceans^27^. Likely, the fact that no metagenomic studies have targeted the aeolian microbiome over the Red Sea thus far is attributable to the very low density of microorganisms in the atmosphere (typically about 10^4^ to 10^6^ cells m^−3^)^28^, about 6 and 8 orders of magnitude lower than in soil (10^12^ to 10^15^ cells m^−3^)^29^ and seawater (10^10^ to 10^13^ cells m^−3^)^29^, respectively. In turn, the amount of DNA typically required to retrieve high-quality metagenomes (~500 ng) would require sampling 6 million m^3^ of atmospheric air^25^ in contrast to much lower sample sizes of seawater (~1 liter)^30^, or soil (~5 g)^31^ required to achieve this DNA content for sequencing. The large air sample size required to recover enough DNA^2,7^ for whole genome shotgun sequencing confounds the analysis of Aeolian microbiomes thus far^27^, as conventional techniques collecting dust on filters would saturate large-sized filters before a fraction of the required volume would be filtered. Indeed, metagenomic databases (e.g. EBI Metagenomics)^32^ lack information on aeolian microbes, despite their interest to unveil the functional strategies underpinning survival under harsh conditions, capabilities for attachment and survive long-distance transport on dust particles, and impacts on organisms, including humans.

Here we provide a first functional genomics assessment of the Aeolian microbiome in the “Global Dust Belt”, with a focus on the genetic traits enabling microbial aeolian lifestyle. We do so by initiating a Gene Catalogue of Aeolian Microbiota (GCAM), based on 10 metagenomes assembled from samples collected over the Red Sea, in the center of the Global Dust Belt. This was enabled by applying of an approach yielding high-quality metagenomes with samples that containing very small DNA amounts (about 5 ng DNA), 100 times smaller than those conventionally required for metagenomics studies, of the aeolian microbiota sampled over the Red Sea. We then interrogate the gene catalogue to detect a parsimonious set of genes coding for functions hypothesized to play a significant role in allowing aeolian microorganisms to survive atmospheric transport.

## Results

All of the metagenomes analyzed were sampled from air masses confined (48 h–120 h back-trajectories) within the global dust belt (Fig. 1a). Samples collected from air masses transported by winds from the prevailing NW direction were characterized by higher dust ranging from 119 to 156 µg m^−3^ and bacterial loads ranging from 109 × 10^3^ to 212 × 10^3^ cells m^−3^ compared to the single sample collected from an air mass transported by winds originating from the SE (5 µg m^−3^ and 288.89 10^3^ cells m^−3^) (Fig. 1b), which is characteristic of these air masses^33^.The DNA content in the ten samples selected for metagenomics analyses ranged from 0.007 to 0.34 ng DNA m^−3^, corresponding to dust concentrations in the samples ranging from 5 to 156 μg m^−3^ (Fig. 1c, Table S2).

An overall total of 68 Giga base-pairs (Gbp) of DNA were sequenced from the ten metagenomes (6.8 Gbp sample^−1^), which produced a total of 2,543,974 redundant coding DNA sequences (CDSs; Table S3). Clustering of the CDS’s at 95% global identity and an overlap of 80% over the shorter sequence length produced 2,370,956 non-redundant CDS sequences, emphasizing the unique genetic repertoire of every metagenomic air sample sequenced here given the low number of redundant genes. The resulting corresponding yield of 30.9 × 10^6^ non-redundant genes per Tera base-pair of DNA sequenced is about three-fold higher than the characteristic yield of metagenomics studies using similar methods^34^ and two-thirds the catalogue of 3.2 × 10^6^ unique predicted genes retrieved from 45 metagenomes sampled across the Red Sea^35^. On a broader context, this corresponds to about 5% of the total number of unique predicted prokaryotic genes resolved within the 243 metagenomes sampled from the global ocean by the TARA Ocean expedition^22^, with a yield of 6.9 × 10^6^ genes per Tb pairs of DNA sequenced. Whereas projects aimed at the global analyses of soil metagenomes are on-going (https://eesa.lbl.gov/soil-metagenome-projects-some-examples/), results are yet to be reported. Moreover, we were unable to find published metagenomes for soils sampled within the Global Dust Belt. Hence, both our global and regional comparisons focus on the ocean and the Red Sea, respectively.

About 73% of non-redundant genes in the GCAM reported here could be reliably assigned to taxonomic entities, 57% were annotated with KEGG orthologs functional role assignments, 49% with gene ontology and 33% were identified as enzymes. Two-thirds (64.9%) of the genes were assigned to bacteria, followed by eukaryotes (5.4%), archaea (1.1%), and viruses (0.69%). In comparison, half of the total coding genes in surface water metagenomes from the Red Sea^36^ (≤100 m) were affiliated with bacteria (59.6%), archaea (4.16%), viruses (6.14%), and eukaryotes (0.46%). Hence, the Aeolian microbiome in the “global dust belt” sampled over the Red Sea is highly enriched in eukaryotic genes (>10-fold higher) compared to the underlying pelagic microbiome. Variability within the sampled aeolian metagenomes was also present. For instance, a sample collected in fall 2016, encompassing an air mass from the NW (KAUST_080) had the highest eukaryotic proportion (21.63%), while KAUST_025, collected in spring 2016 from an air mass from the NW had the lowest (6.5%; Table S6). On the other hand, KAUST_080 was dominated by bacterial genes (22.8%), while KAUST_087, collected in fall 2016 from a NW air mass, was dominated by eukaryotic and viral genes (21.96% and 27.79% respectively). Also, KAUST_091 collected in fall 2016 from a SE air mass was dominated by archaeal genes (23.42%; Table S7).

**Figure 2:**
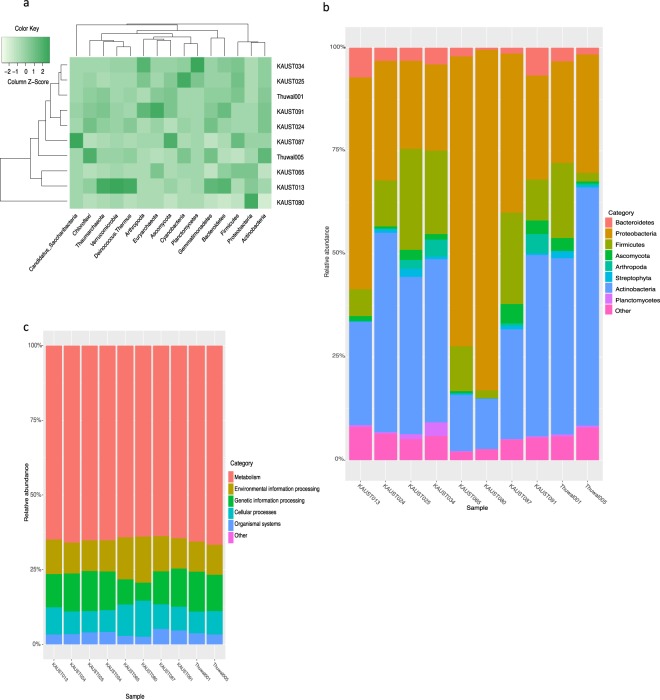
Taxonomical and functional composition in aeolian metagenomes. (**a**) Heat map showing the most abundant phyla in ten aeolian metagenomic data sets using blast and clustering methods. (**b)** Bar chart of the relative abundance of aeolian microorganisms at phylum level in aeolian metagenomes. **(c)** Bar chart of the relative abundance of the biological KEGG pathways in aeolian metagenomes.

At the phylum level, *Ascomycota* and *Arthropoda* genes dominated the sequences assigned as eukaryotic coding genes in the ten aeolian metagenomes, while *Actinobacteria*, *Proteobacteria*, and *Firmicutes* dominated the bacterial sequences, whereas *Euryarchaeota* and *Thaumarchaeota* dominate the archaeal sequences (Fig. 2a,b, Table S5). Thuwal_005, which was collected in spring 2016 from a NW air mass, has a higher abundance of *Actinobacteria* coding genes, while KAUST_080 is dominated by *Proteobacteria*, whereas *Firmicutes* and *Cyanobacteria are the most abundant in* KAUST_025, while KAUST_013, which collected in winter 2015/2016 from an air mass from the NW and KAUST_091 are dominated by *Bacteroides* (Fig. 2a,b). For eukaryotes, *Ascomycota* genes dominate Thuwal_001, which collected in winter 2015/2016 from an air mass from the NE and KAUST_087, while KAUST_034, which collected in spring 2016 from an air mass from the SW and KAUST_091 are dominated by *Arthropoda* (Fig. 2a,b). Under the archaeal domain, *Euryarchaeota* dominates most of the metagenomes, while *Thaumarchaeota* dominates KAUST_013 and Thuwal_005 (Fig. 2a,b).

Based on comparison to public databases such as KEGG, UniProtKB or InterProDB, 630,200 of the genes in the catalogue could not be annotated—that is, roughly 27% of the GCAM could not find a match to any gene throughout BLAST or InterPro. In contrast, only about 22% (390,457 genes) of the gene catalogue (of 1,729,546 non-redundant genes) of pelagic metagenomes from the Red Sea were hypotheticals, suggesting a higher number of novel gene families in GCAM. The taxonomic assignment of sequences to bacterial phyla might be correlated to soil microbiota^20^, as the results show high abundances of *Actinobacteria*, *Bacteroidetes*, *Proteobacteria*, *Firmicutes*, and *cyanobacteria* that mainly inhabit desert soil^37^. Moreover, our findings support the presence of *Gemmatimonadetes* in diverse environmental metagenomes, including air^38^. However, *Thaumarchaeaota* and *Euryarchaeota*, the archaeal assigned sequences appear to be originated from planktonic marine environments^39^. In addition, the high presence of reads assigned to *Bacillariophyta* (71%), diatoms, in a sampling day when a phytoplankton bloom was ongoing in the bearing of incoming wind (Fig. S3) indicates the aerosolization of microbes from seawater to the atmosphere. Taxonomically diverse assemblages of aeolian eukaryotes were detected, including *Ascomycota, Cordate, Arthropoda, Basidiomycota*, and *Microsporidia*, suggesting mixed origins of microbes based on backward trajectories. There was no clear grouping of the communities sampled by locations (offshore and land-sea interface), backward trajectories or sampling season (Fig. S4).

**Figure 3:**
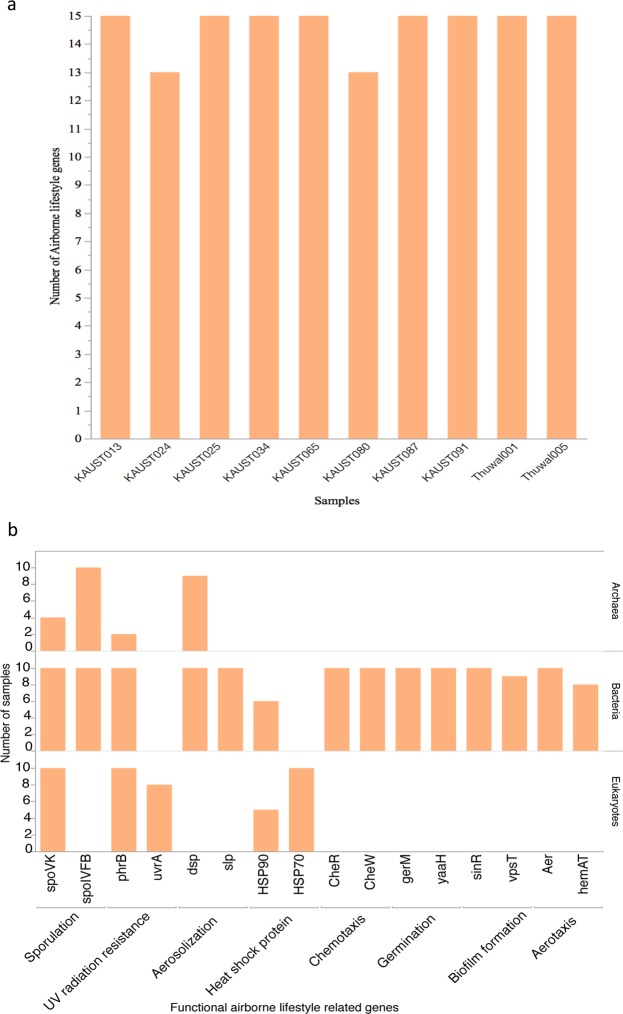
Correlation between counts of metagenomic aeolian samples and the distribution of aeolian lifestyle related genes. (**a**) The occurrence of targeted aeolian lifestyle related genes in aeolian samples. (**b)** The distribution of aeolian lifestyle related genes in aeolian samples per domain.

We used the Dragon Metagenomic Analysis Platform (DMAP)^40^ to further explore the functional assignment of the metagenomics data, in particular, the presence and abundance of aeolian lifestyle-related genes were investigated among the ten metagenomes using their KEGG IDs (Table S4). For robust bioinformatic analysis, DMAP allows filtering genes based on sequence similarity statistics from BLAST based comparison to public databases saved during the annotation process. Here we report results based on percent identity and query coverage cutoffs of 40 and 60, respectively. We specifically explored the presence of genes involved in mechanisms reported to improve survival and transport during the aeolian phase (aerosolization, chemotaxis, aerotaxis, sporulation, germination, biofilm formation, UV radiation resistance, and thermal resistance)^16,41–43^, thereby facilitating the aeolian lifestyle. Profiling of the genes belonging to these biological traits showed that their abundances varied greatly across the ten metagenomes (Fig. 3), but they retained somewhat similar functional profiles of KEGG categories (Fig. 2c). Generally, genes involved in metabolic processes, as well as genes coding for functions facilitating the aeolian lifestyle (e.g. aerosolization), comprised the largest share of the predicted unique genes annotated to functions in the aeolian microbiome (Fig. 2c). Genes encoding potential functions enabling the aeolian lifestyle showed contrasting abundances (i.e. number of copies in a sample) among domains as well as metagenomes sampled from air masses differing in backward trajectories and seasons.

**Figure 4:**
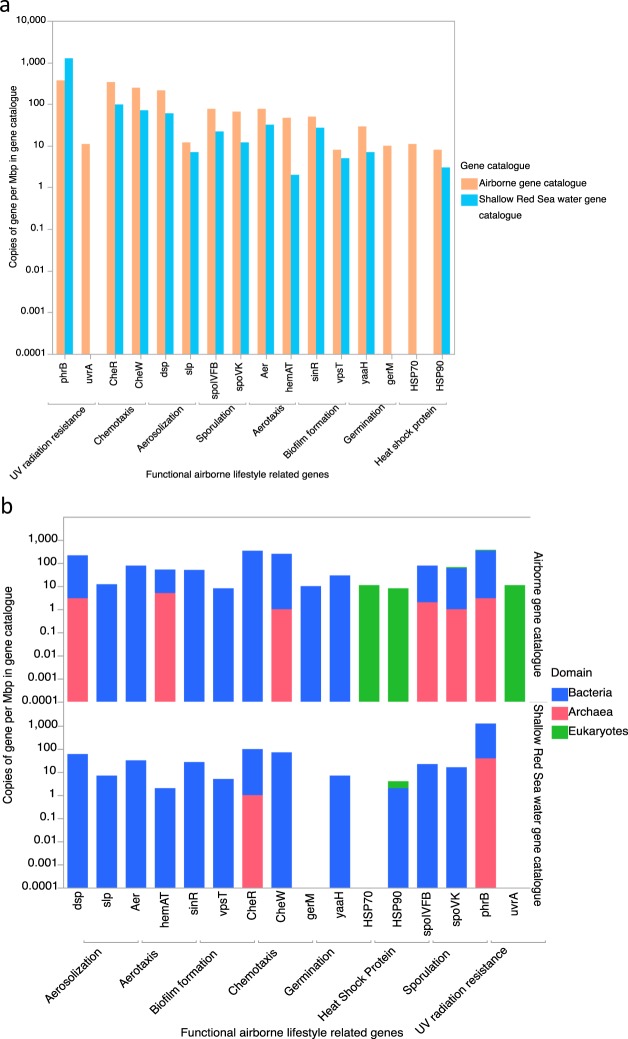
Comparison between read mapping to aeolian gene catalogue and the Red Sea water gene catalogue. (**a**) The total number of aeolian lifestyle related genes in aeolian gene catalogue and the Red Sea water gene catalogue. (**b)** The presence of aeolian lifestyle related genes in the three domains of life (archaea, bacteria, and eukaryotes) in aeolian gene catalogue and shallow Red Sea water gene catalogue.

Importantly, one set of genes, coding for proteins involved in the repair of UV-induced DNA damage (UVrA and phrB)^16^, was present across representative of all three domains of life (Fig. 4b). In contrast, aerosolization related genes were present only in bacteria and archaea. Although their abundance differed—depending on the origin of the sampled air mass, the ubiquity of these genes across domains suggests the capacity to repair UV-induced damage and to aerosolize as fundamental requirements for the aeolian lifestyle. Bacteria contained additional genes coding for other traits putatively enabling the aeolian lifestyle, including chemotaxis (CheW and CheR)^42^, aerotaxis (Aer, hemAT)^44^, germination (gerQ and yaaH)^45^, thermal resistance (HSP70 and HSP90), and sporulation (SpoIVFK and spoVK)^43^. These were particularly enriched in communities sampled from air masses following a NW trajectory. However, sporulation-coding genes were also enriched in bacteria sampled from air masses transported from the SW. Aeolian bacteria were also enriched in genes coding for biofilm formation, facilitating attachment to dust (Fig. 4).

## Discussion

The results presented here provide, by solving the challenge of applying massive sequencing approaches at the low DNA concentration of open-air aerosol samples, a pioneer analysis of the Aeolian microbiome. The few aeolian microbiomes published thus far refers to indoor and built outdoor urban samples^25,26^, which are enriched about 100 times in microorganisms relative to outdoors and are not representative of the microbes transported across the atmosphere, but of those emitted by humans in indoor habitats and cities. Whereas microbiomes have been studied across a range of habitats^46^, including thousands of published metagenomes from soils, ocean, and plant and animal halobionts^27^, our results provide the first metagenomics assessment of the aeolian microbiome and the corresponding gene catalogue. Aeolian microbial metagenomes were rich in unique genes, with a yield of 30.9 × 10^6^ predicted genes per Tera base-pair of DNA sequenced, resulting in a total of 2.37 million non-redundant coding DNA sequences contained in the GCAM. The aeolian communities were dominated with *Actinobacteria*, *Proteobacteria*, and *Firmicutes* of the prokaryotic genes and Ascomycota of the eukaryotes, which is in line with findings in other bioaerosols studies^47–50^. Moreover, these communities were variable, likely a consequence of the diverse sources of the microorganisms present, and highly enriched in eukaryotic genes compared to the underlying seawater surface microbiome. The application of massive sequencing approach revealed that the aeolian microbiome of the Global Dust Belt is a rich source of novel genes sequences given that over half a million predicted genes were not anchored with functions based on current reference databases encompassing gene sequence from diverse biomes. Importantly, our study emphasizes that microbial aeolian lifestyle might be dependent on the prevalence of key genetic features enabling long-range dispersal, resistance to harsh atmospheric conditions and extended non-vegetative periods, which differs significantly from a planktonic lifestyle. In addition, genes encoding proteins involved in repairing UV-induced DNA damage and aerosolization of cells were ubiquitous across samples, and appear as fundamental requirements for the aeolian lifestyle. That is based on the fact that the Red Sea atmosphere is exposed to intense UV radiations that causing DNA damage. Moreover, the high atmospheric dust concentrations in this region plays an important factor in either scatter or absorb the UV radiation, where the attached microbes to the dust particles could be affected and genes such as UVrA are needed to repair the UV-induced damage^51–54^. Although no metagenomes of soil-borne microbes sampled in the global dust belt are available, we compared the relative abundance of genes selected with those in soil metagenomes sampled in Alaska^55^ and Australia^56^. We observed that the relative abundance of Chemotaxis, HSP, Sporulation, and UV resistance genes in soil metagenomes were depleted in the soil samples compared relative to the aeolian bacteria metagenomes reported here. However, we caution that this comparison is weakened by the fact that the soils available are unlikely to act as sources for the airborne bacteria sampled here and the limited number of airborne metagenomes analyzed here (n = 10) precludes robust comparisons.

Our metagenomics analyses of Aeolian microbiome demonstrate the enrichment of particular functional traits likely associated with the aeolian lifestyle, many of which are overrepresented relative to ambient microbial communities in pelagic waters of the Red Sea. In particular, sporulation genes were greatly enhanced in the aeolian microbiome compared to Red Sea plankton community, along with chemotaxis, germination, and heat-shock protein genes—all facilitating survival microbes in harsh environments^57^, and in turn, long residence time and transport of the aeolian microbiome. The lack of desert soil metagenomic microbiomes precluded comparison of our datasets with metagenomes from soils that can be putative sources of organisms to the atmosphere in the Global Dust Belt. Aerosolization, aerotaxis, biofilm formation, and UV-damage DNA repair genes were comparable, in standardized abundance, with Red Sea metagenomes, further suggesting the Red Sea to be a likely source of microorganisms fit for aeolian lifestyles.

Our results do not suggest evidence of major differences in metagenomics composition of the microbes, for the targeted genes, depending on season, although the small sample size (e.g. only 1 metagenome collected in summer), involves low power in detecting such differences. Whereas no prior study of functional genomics of Aeolian dust is available, studies of community structure, using amplicon sequences, do not consistently report changes in community structure. For instance, Li *et al*.^58^ report seasonal differences in community structure of Aeolian communities sampled in China, whereas Park *et al*.^59^ report no seasonal difference over Japan.

These findings not only have implications for conservation genetics and forensic microbiology but also human health, as they imply that microbial communities that settle on sand storms carry functional traits beneficial for their large-scale dispersal, that may propagate microorganisms interacting with humans and animals, such as pathogens, across vast distances.

## Materials and Methods

### Samples collection

For decontamination procedure, new clean filters were combusted before sampling at 200 °C for 24 h, and pre-conditioned at ambient temperatures and relative humidity (21 °C and 60% RH) before use. To grip the filters, sterilized tweezers were used. Filters were placed in filter holders (circular filter holder for 15 cm diameter filters) (MCV SA, Collbató, Barcelona, Spain) (http://www.mcvsa.com/Productes/Atm%C3%B3sfera/CaptadordealtovolumenMCVCAVAmb/tabid/114/Default.aspx) that have been preserved in 4% HCl for 12 h. Filters and filter holders set were put inside clean plastic zip bags and transported to the sampler. Figure S2 shows the control filter and the cleaning protocol of the sampling equipment. Regular sampling of Total Suspended Particulates (TSP) was performed using automatic sequential high-volume samplers (MCV SA, Collbató, Barcelona, Spain) (http://www.mcvsa.com/Productes), equipped with TSP cut off inlets at a flow rate of 20 m^3^ hr^−1^ over periods of 24 hours to one week. Air was sampled through the inlet by means of an in-built pump. The ambient air was filtered to collect the suspended particles on quartz fiber filters (Whatman™ 1810-150 Acid Treated TCLP Filter for EPA Method 1311 with Low Metals, diameter: 15 cm, pore size: 0.6–0.8 µm). The high-volume sampler on board the research vessel was equipped with a weather vane, which would switch off the pump and thus cease sampling immediately whenever the sampler was downwind of the ship’s exhaust. The mass concentration of TSP was determined by weighing the filters before and after sampling and expressed as µg per m^3^. Aeolian dust samples were collected at two locations: a land-ocean interface and an offshore regions of the Red Sea from December 2015 to November 2016 (Table. S1). A new clean filter was used as control. However, dust loads on control filters were below detection limit (by weight) and had, accordingly, too little materials to support sequencing (Fig. S2).

### Backward trajectory calculation

Multiple models allow the analysis of air trajectory at a certain time. In this study we used The HYSPLIT model (available online at https://ready.arl.noaa.gov/), which is a complex atmospheric system for computing simple air particle trajectories as well as complex transport, dispersion and deposition simulations using archived meteorological data. The meteorological data obtained through the Real‐time Environmental Applications and Display System and archived in the Global Data Analysis System (GDAS1) to calculate the source of the air being sampled backward up to 120 hours and on three height levels (200 m, 300 m, and 800 m) using metrological data of the archived analysis (Fig. S1). Sample backward trajectories were calculated for 120 hours counted since the end of sampling. As the average sampling time was 3.3 days, this includes 2 days prior to the onset of sampling. However, in the offshore samples that was collected over 5 days, the backward trajectory starts on the first day of sampling.

### DNA extraction

Total DNA was isolated from dust filters samples for whole-genome sequencing using phenol-chloroform extraction protocol. Briefly, a quarter of 150 mm Whatman fiber filters (GE HealthCare Bio-Sciences, Pittsburgh, PA, USA) were cut into small stripes and 5 ml of lysis buffer (prepared with 0.5 M EDTA, 1 M Tris-HCl (pH 8) and NaCl) was added to the tubes containing ca. 100 mg of 0.1 mm glass beads (BioSpec Products, Bartlesville, OK, USA) and ca. 100 mg of 0.1 mm zirconia beads (BioSpec Products, Bartlesville, OK, USA). To each tube, of 1 mg/ml lysozyme (Sigma-Aldrich, St. Louis, MO, USA) was added and mixed. By inverting every 15 minutes, the tubes were incubated at 37 °C for 30 minutes. After incubation, 205 μl of 20% SDS and 10 μl of proteinase K (QIAGEN, Valencia, CA, USA Cat. No. 19133) were added and mixed thoroughly for incubation at 55 °C for 2 hours with inverting the tubes every 30 minutes. Then, 5 ml of phenol-chloroform-isoamyl alcohol (Sigma-Aldrich, St. Louis, MO, USA) was added followed by centrifugation at maximum speed for 10 minutes. After spinning, white interference layers were visible and the supernatants were transferred to fresh microcentrifuge tubes leaving the interference behind. To remove the phenol, 5 ml of chloroform-isoamyl alcohol (AppliChem, GmbH, Darmstadt, DE) was added to each tube and centrifuged for 5 minutes in a microcentrifuge. The supernatants were transferred to 10,000 MW cutoff Amicon Ultra centrifugal filters (Millipore, Burlington, MA, USA) and centrifuged in a swinging rotor for 15 minutes. Filtrates in the lower tubes were discarded and 5 ml of 10 mM Tris-HCl was added to each tube and centrifuged until the volume reduced to <250 μl. For the second time, filtrates were discarded and 10 mM Tris-HCl was added to each tube to reach 250 μl following mixing. The concentrated volumes in the upper tubes containing the extracted DNA were transferred to clean 2 ml Eppendorf tubes and quantification of DNA was done using Qubit dsDNA HS (High Sensitive) Assay Kit (Thermo Scientific, Invitrogen, Carlsbad, CA, USA) and Promega® GloMax-Multi Detection System (Promega Corporation, Madison, WI). For the control protocol, a new clean filter was used as the negative control, where DNA concentration was below detection limit. The dust filters that used in the study of Yahya *et al*.^33^ were used as positive control, which had been confirmed to contain dust and microbial cells through microscopic identification (Fig. S2a,b).

### Metagenomic library preparation, sequencing, and analyses

Ovation Ultralow library systems V2 (NuGEN, San Carlos, CA, USA) were used to prepare the aeolian metagenomic libraries due to the typically low DNA concentration present in atmospheric dust samples. This kit produces high-quality libraries for next-generation sequencing (starting from 10 pg of DNA) without the need for pre-amplification and decreasing the PCR artifacts. Briefly, genomic DNA was normalized to 5 ng (which was the minimum DNA present in one sample) in a final volume of 120 µl and was sheared to an average size of 400 bp with an M220 ultrasonicator (Covaris, Woburn, MA, USA). Sheared DNA samples were used for paired-end indexed library construction using Ovation Ultralow library systems V2 (NuGEN, San Carlos, CA, USA), according to the manufacturer instructions. Most of the fragments were recovered because no size selection was applied. Thirteen PCR cycles and Illumina adapter-specific primers amplified the DNA fragments. AMPure XP beads (0.8×; Beckmann Coulter Genomics, Brea, CA, USA) were used to purify the libraries. The quality and concentration of amplified products were assessed by Bioanalyzer (Agilent Technologies, Santa Clara, USA), which showed that all samples had a similar peak shape, and Qubit (Thermo Scientific, Invitrogen, Carlsbad, CA, USA) which showed that all the samples were in a similar concentration range. The indexed libraries were pooled in equimolar concentration and subjected to Illumina HiSeq 4000 platform deep sequencing on one lane using Hiseq 3000/4000 SBS reagent 300-cycle kit (Illumina, Inc., Alliance Global FZ, Dubai, UAE) and bidirectional sequencing of 150 bp. Library preparation, multiplexing, and deep sequencing were performed at the Bioscience Core Lab facility at King Abdullah University of Science and Technology, Saudi Arabia. Raw read sequences were quality-trimmed while removing adaptor sequences, using Trimmomatic v0.323^60^ with the following parameters: ILLUMINACLIP::4:30:10 LEADING:20 TRAILING:20 SLIDINGWINDOW:4:20 MINLEN:60. The internal sequencing standard PhiX 174 was subsequently removed by mapping the quality-trimmed reads against the PhiX 174 genome using Bowtie2 v2.2.45^61^ with default settings. At each stage, the quality of read sequences was assessed using FASTQC (http://www.bioinformatics.babraham.ac.uk/projects/fastqc/).

The resulting high-quality paired-end reads for each dataset (*n* = 10 samples) were assembled independently with metaSPAdes v3.9.0^62^ using a kmer range of 21 to 127 while employing the error-correction mode and preset metagenomic options. The assembled contigs were then filtered to a minimum length of 500 bp followed by gene prediction using MetageneMark v3.38^63^. We retained protein-coding genes with a minimum length of 100 bp, yielding a total of 2,543,974 redundant genes, averaging (±SD) 254,397 ± 85,976 genes per sample (*n* = 10). Table S3 summarizes the general statistics of read sequences, the assembled contigs, and predicted genes across the different samples.

These 2.54 million genes were clustered to generate a non-redundant gene catalogue using CD-HIT (95% nucleotide identity and 80% overlap of the shorter sequence), resulting in the Gene Catalogue of Aeolian Microbiome (GCAM) with 2,370,956 non-redundant gene sequences. The resultant representative gene sequence clusters were subsequently functionally and taxonomically annotated using the online server DMAP^40^ by applying a minimum blast bit-score of 60% for the functional assignment using KEGG^64^.

A gene abundance matrix was generated by mapping the error-corrected paired-end reads against the Aeolian microbiome gene catalogue using Bowtie2^61^ with default settings. The resultant mapped read counts per gene (and sample) were normalized into a common metric of gene abundance—that is, fragments per kilobase of exon per million fragments mapped (FPKM) using Cufflinks and Cuffdiff^65^. Both the annotated gene catalogue and abundance matrix were then placed in DMAP for further interrogation including community and gene profiling for metabolic pathways of interest.

### DMAP based annotation and sample comparison

DMAP has two modules one for annotation and another for sample comparisons, see DMAP documentation at http://www.cbrc.kaust.edu.sa/aamg/docs/DMAP_Documentation.html. Extended taxonomic and functional annotations of GCAM produced by DMAP annotation module are indexed for interactive visualizations and comparisons in DMAP compare module developed by extending the functionality of Metagenomic Reports (MetaRep) software^66^.

Taxonomic assignments for genes are carried out using high throughput BLASTp comparisons to Universal Proteins Knowledgebase (UniProtKB) considering both best blast hit and the least common ancestor approach (LCA). The LCA based taxonomic assignment results are loaded to DMAP comparison module for interactive interrogation. Functional role assignments for genes are carried out using BLASTp to KEGG ortholog (KO) assigned sequences from KEGG database. KEGG orthologs are linked to KEGG enzymes, modules and pathways. In cases where no functional role assignments are available from KEGG, generic gene descriptions are obtained from UniProtKB BLASTp results. Gene Ontology and signature functional domain information is obtained through InterProscan analysis. A final gene information table produced based on DMAP based annotations is indexed and deposited to DMAP Compare module that links Taxonomy, KEGG Ortholog, Enzymes, Gene Ontology Pathways identifiers to their parent-child hierarchies for a deeper interactive analysis.

Gene catalogue information table is submitted to DMAP compare module considering a weight of 1 or 0 to denote gene presence or absence information. Gene information tables are produced for each sample by extracting sample specific gene abundance estimate from the gene abundance matrix alongside annotations of corresponding genes from the common gene catalogue. These sample specific gene information tables are indexed and deposited to DMAP compare module similar to a gene catalogue but considering sample specific gene abundance estimates as weight.

The compare function in DMAP comparison module allows users to select several samples and see comparative results as tables or visualizations such as heatmaps, bar graphs, Multi-Dimensional Scaling (MDS) and other interactive heatmaps, see DMAP documentation for a full overview and examples. For taxonomic profiling of samples, users can select a filter either 16S genes or universal single-copy genes (SCGs) from the list of filters provided. In case of samples with only protein-coding genes, it is best to select one of the filters from DMAP provided Universal, Prokaryotic or Eukaryotic single copy genes filters. It may be appropriate to initially look at all SCGs based results for selected samples and later choose a more appropriate one SCG as a proxy to 16S gene like profiling of samples. Users can increase or decrease the fold change control to clearly find any profound patterns in the results.

An exciting feature of DMAP compare module is the ability to compare samples based on groups of genes required to activate a complete pathway module. Predefined groups of necessary or alternative KOs are compared with sets of KOs available from selected samples and an interactive heatmap is produced showing white cells for incomplete modules and red for complete modules across samples being compared. Clicking on a cell leads to KEGG Pathway module diagram showing sample specific genes highlight in red to describe the completeness of a module for a sample in question.

## Supplementary information

Aalismail et al Supp info SREP-19-17724A

## Data Availability

All sequence data generated by this study have been submitted to the ENA European Nucleotide Archive under the accession number PRJEB31563. Gene Catalog and related samples with annotation are available for public access through DMAP project 58 (click on public access at http://www.cbrc.kaust.edu.sa/dmap to see project 58).
