# Supplementary material for: Functional metagenomic analysis of dust-associated microbiomes above the Red Sea
Source: Sci Rep. 2019 Sep 24;9:13741. doi: 10.1038/s41598-019-50194-0 (PMC6760216; doi:10.1038/s41598-019-50194-0)
Supplement: Supplementary file 1 — Aalismail et al Supp info SREP-19-17724A [file 41598_2019_50194_MOESM1_ESM.pdf]

Functional metagenomic analysis of dust-associated microbiomes above the Red Sea

Nojood A. Aalismail[1†], David K. Ngugi[2], Rubén Díaz-Rúa[1], Intikhab Alam[3], Michael Cusack[1], and Carlos M. Duarte[1]

1. Red Sea Research Centre (RSRC) and Computational Bioscience Research Center (CBRC), King Abdullah University of Science and Technology (KAUST), Thuwal 23955, Saudi Arabia

2. Department of Microorganisms, Leibniz Institute DSMZ - German Collection of Microorganisms and Cell Culture, Inhoffenstrasse 7 B38124 Braunschweig, Germany

3. Computational Bioscience Research Center, King Abdullah University of Science and Technology, Thuwal 23955, Saudi Arabia

[†] Corresponding author: E-mail: Nojood.aalismail@kaust.edu.sa, Tel: +966590592006

Supplementary information:

From December 2015 to November 2016, 121 dust samples were collected until the filter was clogged, which involved sampling periods ranging from 24 h, during dust storms, to 1 week, when dust loads were lowest. Samples were subsequently selected for whole-genome shotgun sequencing on the basis of their DNA concentration, since dust DNA loads are much lower than those typically required for shotgun sequencing, which range from 2 to 25 ng/µl with 15 µl as

minimum sample volume for Illumina sequencing (https://dnatech.genomecenter.ucdavis.edu/illumina-library-sequencing/). The minimum of 5 ng DNA per sample used as cut-off that was obtained in about half (N = 58) samples, where DNA concentrations in the elution buffer extracts obtained ranged from 0.07 ng/µl to 0.5 ng/µl, about 30 to 50 fold lower than the concentrations typically required for Illumina sequencing. Then we assessed the backward air trajectories of the air masses sampled using the HYSPLIT model (Fig.S1), and classified the samples into distinct air mass sources (3 primary sources, Europe, N. Africa, and The Arabian Peninsula). Each of the sources was considered to be selected for metagenomic analysis, resulting in half (N = 27) of the samples remaining as candidates for analysis. We then classified these 27 dust samples into sampling season and location (land-sea interface vs. offshore, depending on whether they were sampled on the harbor of King Abdullah University of Science and Technology or sampled on the research vessel during a cruise). This resulted in a final selection of 10 samples for sequencing, including three samples from each season, except for only one in summer, when DNA loads tended to be very low (and only one sample met the minimum DNA required), sampled in the land-sea interface (n = 8) and offshore (n = 2), as these were the only samples collected offshore that had sufficient DNA loads to allow shot-gun sequencing. The sampling duration for the 10 samples selected for sequencing ranged from 2 days to 5 days, with an average of 3.3 days, with the offshore samples collected over 5 days (Table. S1)

Table S1. Sampling metadata. Airborne samples collection dates, locations, and metrological data (www.wunderground.com).

Sample	Sampling season	Latitude	Longitude	Sampling location	Sampling duration	Wind backward trajectory	Wind speed (km/h)	Average temperature (°C)	Average UV index	Average relative humidity
**KAUST013**	Winter2015/2016	22.41	39.14	Land-sea interface	2 days	NW (Africa)	14	26	6	52
**Thuwal001**	Winter2015/2016	25.33	37.03	Offshore	5 days	NE (Europe)	8	24	8	45
**KAUST024**	Winter2015/2016	22.41	39.14	Land-sea interface	2 days	W (Africa)	12	27	8	58
**KAUST025**	Spring2016	22.41	39.14	Land-sea interface	3 days	NW (Africa)	16	27	6	66
**Thuwal005**	Spring2016	27.23	34.95	Offshore	5 days	S (Africa)	35	32	6	43
**KAUST034**	Spring2016	22.41	39.14	Land-sea interface	2 days	SW (Africa)	8	34	8	36
**KAUST065**	Summer2016	22.41	39.14	Land-sea interface	2 days	NW (Africa)	8	34	8	36
**KAUST080**	Fall2016	22.41	39.14	Land-sea interface	3 days	NW (Europe)	12	32	8	64
**KAUST087**	Fall2016	22.41	39.14	Land-sea interface	2 days	E (Arabian Peninsula)	5	30	8	53
**KAUST091**	Fall2016	22.41	39.14	Land-sea interface	5 days	SE (Arabian Peninsula)	6	28	8	53

Table S2. Air sampling data. Total suspended particles concentration in volume of sampled air and DNA concentration.

Sample	TSP (µg/m^3)	DNA amount (ng)	DNA conc. (ng/µl)	Air volume (m^3)	TSP (µg)	DNA in TSP (ng/m^3)
**KAUST013**	156	51.54	0.257	2519	392600	0.020
**KAUST024**	151	103.45	0.517	300	45400	0.344
**KAUST025**	96	23.58	0.117	3360	323700	0.007
**KAUST034**	132	49.67	0.248	322	42600	0.154
**KAUST065**	119	20.79	0.103	796	95100	0.026
**KAUST080**	138	17.74	0.088	816	112500	0.021
**KAUST087**	100	78.93	0.394	959	95700	0.082
**KAUST091**	5	46.36	0.231	547	2530	0.084
**Thuwal001**	57	5.6	0.078	1901	108100	0.008
**Thuwal005**	36	34.81	0.174	2026	72600	0.017

Table S3. General features of the megadomes analyzed in this study including the number of contigs and the predicted number of genes.

	Statistics of the assembled contigs (nucleotide)						Predicted protein-coding genes (nucleotides)			
**Sample**	**#Contigs**	**Total length (bp)**	**Aver. length**	**Max length**	**GC %**	**N^{50} (bp)**	**#Counts**	**Total length (bp)**	**Aver. length**	**Max length**
KAUST013	218,063	158,933,053	729	52,936	60.5	79,466,902	294,182	116,813,553	397	9,042
KAUST024	197,725	139,143,570	704	133,755	62.5	69,572,202	267,085	101,693,760	381	14,358
KAUST025	149,466	107,206,087	717	42,686	52.8	53,603,204	180,770	67,833,696	375	4,884
KAUST034	255,968	205,068,274	801	137,074	53.4	102,534,315	324,173	129,553,656	400	8,742
KAUST065	174,578	170,464,938	976	927,321	55.6	85,232,897	269,778	128,913,198	478	15,450
KAUST080	41,334	72,136,022	1,745	1,336,562	61.4	36,070,781	95,957	60,661,041	632	17,688
KAUST087	128,819	122,505,462	951	674,060	52.6	61,253,423	178,141	83,631,729	469	13,026
KAUST091	383,702	297,320,506	775	40,284	58.2	148,660,814	405,223	145,839,480	360	4,335
Thuwal001	192,068	136,134,692	709	42,729	59.4	68,067,355	250,319	94,220,103	376	5,235
Thuwal005	218,006	148,431,308	681	42,484	66.0	74,216,032	278,346	105,412,272	379	4,881
**TOTAL**	**1,959,729**	**1,557,343,912**					**2,543,974**	**1,034,572,488**		
**Average**	**195,973**	**155,734,391**					**254,397**	**103,457,249**		
**SD**	**88,742**	**61,344,441**					**85,976**	**27,667,737**		

Table S4. Targeted Airborne lifestyle functional genes.

Function	KEGG ID	Gene definition	Gene
**Aerosolization**	K07285	outer membrane lipoprotein	slp
	K04047	starvation-inducible DNA-binding protein	dsp
**Aerotaxis**	K03776	aerotaxis receptor	Aer
	K02491	heam-based aerotactic trancducer	hemAT
**Chemotaxis**	K03408	purine-binding chemotaxis protein	CheW
	K00575	chemotaxis protein methyltransferase	CheR
**Germination**	K06298	germination protein	gerM
	K06306	spore germination protein	yaaH
**Sporulation**	K06402	stage IV sporulation protein FB	spoIVFB
	K06413	stage V sporulation protein K	spoVK
**UV radiation resistance**	K01669	deoxyribodipyrimidine photo-lyase	phrB
	K21249	UV radiation resistance-associated gene protein	uvrA
**Heat resistance**	K09487	heat shock protein 90kDa beta	HSP90
	K09489	heat shock 70kDa protein 4	HSP70
**Biofilm formation**	K20918	positive regulator of biofilm formation	vpsT
	K19449	master regulator for biofilm formation	sinR

Table S5. Relative abundance of phylum genes, differentially present in the ten airborne metagenomes.

Phylum	KAUST 013	KAUST 024	KAUST 025	KAUST 034	KAUST 065	KAUST 080	KAUST 087	KAUST 091	Thuwal 001	Thuwal 005
Bacteroidetes	0.0636	0.0277	0.0265	0.0343	0.0225	0.0057	0.0126	0.05	0.0288	0.0138
Chlorobi	0.0001	0.0001	0	0.0001	0	0	0	0.0001	0.0001	0.0001
Cyanobacteria	0.007	0.0067	0.0118	0.0088	0.0025	0.0007	0.0022	0.0043	0.0056	0.0075
Proteobacteria	0.411	0.2309	0.1604	0.1647	0.6243	0.8079	0.2827	0.1686	0.1977	0.221
Firmicutes	0.0514	0.0884	0.1779	0.147	0.1001	0.0205	0.1791	0.0648	0.1387	0.018
Deinococcus-Thermus	0.0084	0.004	0.0017	0.0022	0.0002	0	0.0002	0.0022	0.0021	0.0035
Bacillariophyta	0.0013	0.0001	0	0	0	0	0.0003	0	0	0
Chlorophyta	0.0004	0.0001	0.0002	0.0001	0.0001	0	0.0009	0	0.0001	0.0001
Chytridiomycota	0	0	0.0001	0.0001	0.0001	0	0.0001	0.0001	0.0001	0
Ascomycota	0.0122	0.0073	0.0221	0.0126	0.0059	0.0005	0.0434	0.026	0.0291	0.0049
Basidiomycota	0.0003	0.0003	0.0009	0.0003	0.0005	0.0001	0.0048	0.0003	0.0007	0.0003
Apicomplexa	0.0006	0.0019	0.0019	0.002	0.002	0.0004	0.0138	0.0007	0.0016	0.0013
Porifera	0	0	0	0	0	0	0	0.0001	0	0.0001
Cnidaria	0	0	0	0.0001	0	0	0.0001	0.0001	0	0
Platyhelminthes	0	0	0.0001	0.0001	0	0	0.0001	0.0002	0.0001	0
Nematoda	0.0033	0.0034	0.0027	0.0025	0.0015	0.0006	0.0065	0.0028	0.0028	0.0029
Annelida	0	0	0	0	0	0	0	0.0001	0	0
Mollusca	0	0	0.0001	0.0001	0	0	0.0002	0.0003	0	0.0001
Arthropoda	0.001	0.0022	0.0191	0.035	0.0013	0.0003	0.0104	0.0383	0.0015	0.0077
Brachiopoda	0	0	0	0	0	0	0	0.0001	0	0
Echinodermata	0	0.0001	0.0001	0.0002	0.0001	0	0	0.0002	0	0
Crenarchaeota	0	0	0	0	0	0	0	0.0001	0	0
Euryarchaeota	0.0011	0.0042	0.0023	0.0037	0.0051	0.0005	0.003	0.0114	0.003	0.0013
Fusobacteria	0	0	0	0	0	0	0.0001	0	0	0
Streptophyta	0.0033	0.0099	0.0213	0.0073	0.0057	0.0009	0.0109	0.0048	0.0182	0.0061
Nitrospirae	0.0005	0.0004	0.0002	0.0006	0.0001	0.0001	0.0001	0.0003	0.0003	0.0005
Acidobacteria	0.0019	0.0018	0.0011	0.003	0.0002	0.0001	0.0003	0.0015	0.0016	0.0018
Candidatus Marinimicrobia	0	0.0001	0.0001	0.0002	0.0001	0	0	0	0	0.0001
Fibrobacteres	0	0	0	0	0	0	0	0	0.0001	0
Candidatus Omnitrophica	0.0001	0.0001	0.0001	0.0002	0	0	0	0.0001	0.0001	0.0001
Candidatus Aminicenantes	0.0001	0.0001	0.0001	0.0001	0	0	0	0.0001	0.0001	0.0001
Armatimonadetes	0.0004	0.0003	0.0001	0.0003	0	0	0	0.0002	0.0002	0.0003
Candidatus Latescibacteria	0	0	0	0.0001	0	0	0	0	0	0

**Elusimicrobia**	0.0001	0.0001	0	0.0001	0	0	0	0	0	0.0001
**Verrucomicrobia**	0.012	0.001	0.0013	0.0016	0.0003	0.0001	0.0003	0.0008	0.0009	0.0012
**Candidatus Saccharibacteria**	0	0	0	0	0	0	0.0024	0.0002	0	0
**Gemmatimonadetes**	0.0093	0.0101	0.0051	0.0042	0.0004	0.0001	0.0005	0.0051	0.0069	0.0085
**Candidatus Hydrogenedentes**	0.0001	0.0001	0.0001	0.0001	0	0	0	0.0001	0.0001	0.0001
**Chloroflexi**	0.0049	0.0073	0.0032	0.0035	0.0004	0.0002	0.0005	0.0047	0.0053	0.0093
**Actinobacteria**	0.2049	0.3854	0.29	0.3056	0.133	0.1201	0.2127	0.298	0.3427	0.4316
**Planctomycetes**	0.0049	0.0043	0.0106	0.0297	0.0016	0.0004	0.0014	0.0027	0.0054	0.0049
**Spirochaetes**	0.0002	0.0003	0.0002	0.0003	0.0002	0	0.0003	0.0002	0.0005	0.0002
**Chlamydiae**	0.0007	0.0018	0.0022	0.0016	0.0014	0.0002	0.0123	0.0007	0.0011	0.0012
**Lentisphaerae**	0.0001	0.0001	0	0.0001	0	0	0	0.0001	0.0001	0.0001
**Candidatus Poribacteria**	0	0	0.0001	0.0002	0	0	0	0	0	0.0001
**Synergistetes**	0	0.0001	0	0.0001	0	0	0	0.0001	0.0001	0
**Tenericutes**	0	0.0001	0.0032	0.0001	0.0001	0	0.0003	0.0001	0	0
**candidate division NC10**	0.0001	0.0001	0	0.0001	0	0	0	0.0001	0.0001	0.0001
**Thaumarchaeota**	0.0068	0.002	0.0008	0.0003	0	0	0.0002	0.0023	0.0022	0.002
**Ignavibacteriae**	0.0001	0.0001	0.0001	0.0002	0	0	0	0.0001	0.0001	0.0001
**Nitrospinae**	0.0001	0.0001	0	0.0001	0	0	0.0001	0	0.0001	0.0001
**candidate division Zixibacteria**	0	0.0001	0	0.0003	0	0	0	0	0	0.0001
**Candidatus Rokubacteria**	0.0004	0.0004	0.0002	0.0005	0.0001	0	0	0.0003	0.0003	0.0004
**Candidatus Woesearchaeota**	0	0.0001	0.0003	0.0013	0.0002	0	0.0001	0	0	0.0001
**Candidatus Tectomicrobia**	0.0002	0.0002	0.0001	0.0002	0	0	0	0.0001	0.0002	0.0002
**Candidatus Eisenbacteria**	0	0	0	0.0001	0	0	0	0	0	0
**Candidatus Handelsmanbacteria**	0.0001	0.0001	0	0.0001	0	0	0	0.0001	0.0001	0.0001
**Rhodothermaeota**	0.0001	0.0001	0.0001	0.0001	0	0	0	0.0001	0.0001	0.0001
**Mucoromycota**	0.0001	0.0002	0.0005	0.0001	0.0001	0	0.0002	0.0002	0.0002	0.0001
**Balneolaeota**	0	0.0003	0.0001	0.0001	0	0	0	0.0001	0	0

Table S6. Relative abundance of annotated reads in the ten airborne metagenomes at domain level.

Domain	KAUST013	KAUST024	KAUST025	KAUST034	KAUST065	KAUST080	KAUST087	KAUST091	Thuwal001	Thuwal005
**Bacteria**	0.9348	0.9295	0.8785	0.8832	0.9592	0.9876	0.8422	0.8388	0.8974	0.9339
**Archaea**	0.0101	0.008	0.0044	0.0068	0.0059	0.0006	0.0039	0.02	0.0066	0.0047
**Eukaryotes**	0.0303	0.0333	0.0908	0.0786	0.0202	0.0034	0.1171	0.1136	0.0701	0.0324
**Viruses**	0.0007	0.0031	0.0059	0.0112	0.008	0.0047	0.0189	0.0013	0.0026	0.0028

Table S7. Relative abundance of total number of genes per domain present in the ten airborne metagenomes

Domain	KAUST013	KAUST024	KAUST025	KAUST034	KAUST065	KAUST080	KAUST087	KAUST091	Thuwal001	Thuwal005
**Bacteria**	0.7959	0.791	0.7172	0.7249	0.8974	0.959	0.7308	0.6193	0.7552	0.7416
**Archaea**	0.0086	0.0068	0.0036	0.0056	0.0055	0.0006	0.0033	0.0147	0.0056	0.0037
**Eukaryotes**	0.0258	0.0283	0.0741	0.0645	0.0189	0.0033	0.1016	0.0838	0.059	0.0258
**Viruses**	0.0006	0.0027	0.0048	0.0092	0.0075	0.0046	0.0164	0.001	0.0022	0.0023

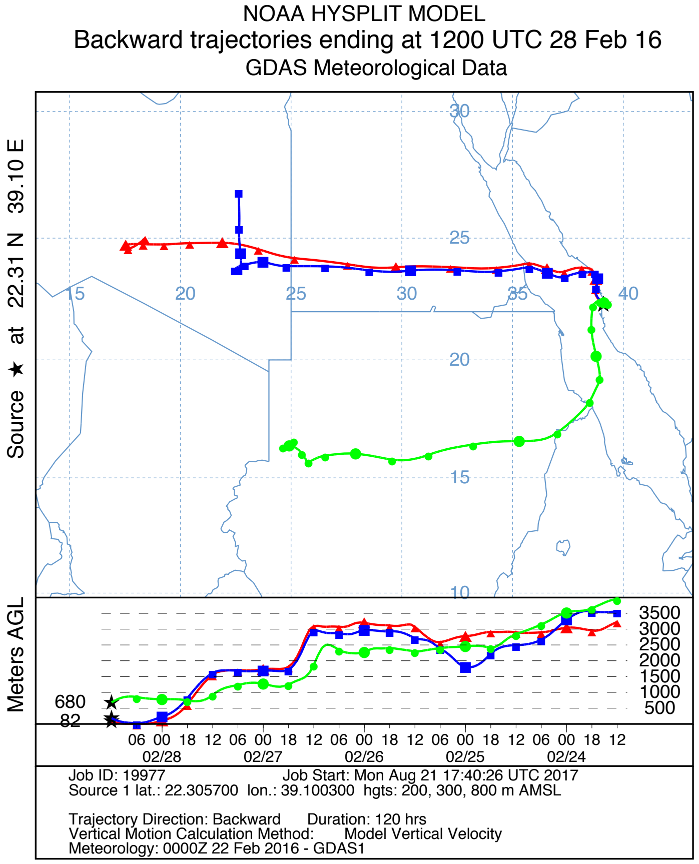

Figure S1. Example of the generated metrological data by HYSPLIT model, using parameters of three height levels: 800m, 300m, and 200m and duration: 120h.

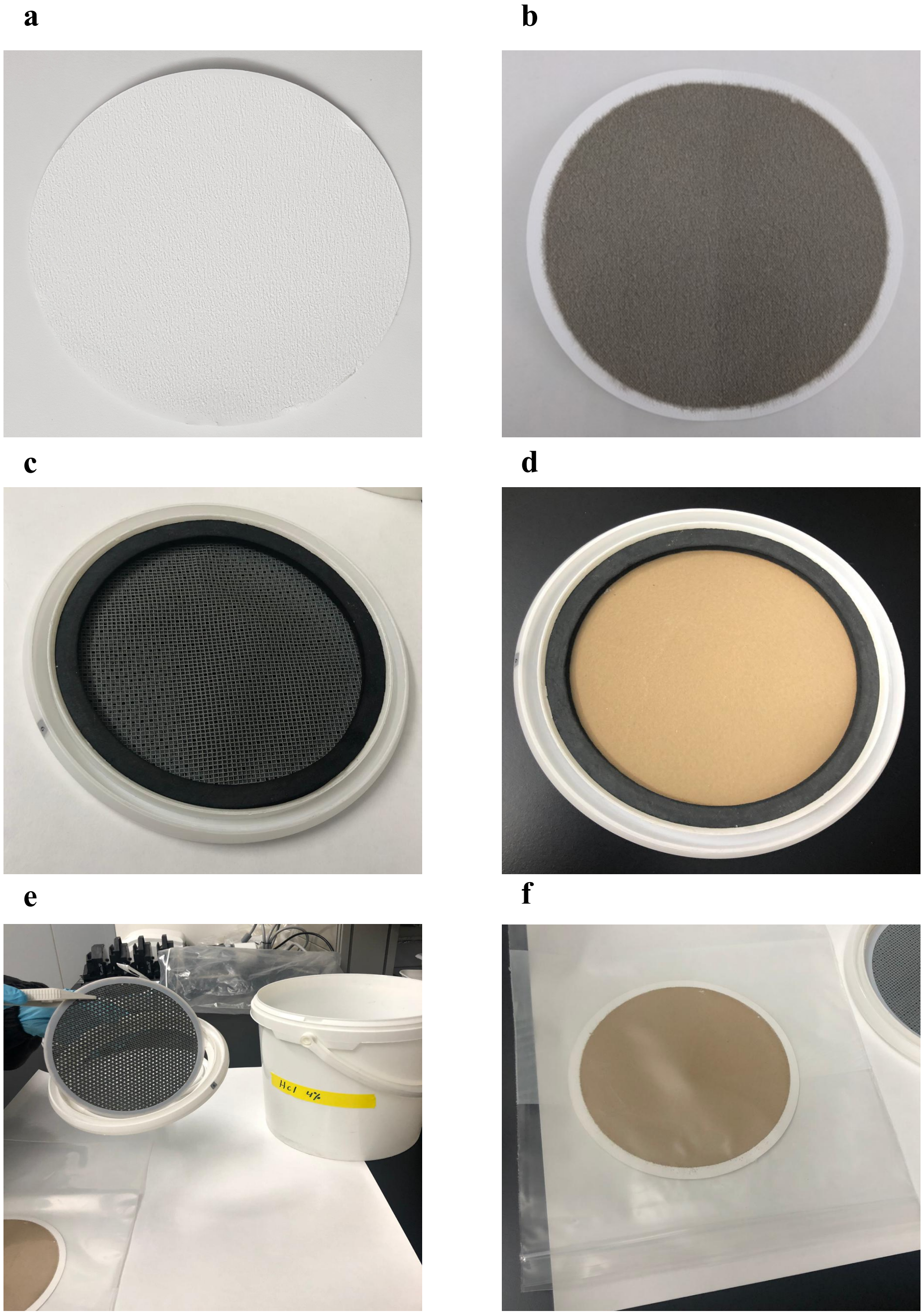

Figure S2. Filters and filter holder images. **a.** New clean filter used as a negative control. **b.** Filter full of airborne dust after sampling used as a positive control. **c.** Empty filter holder. **d.** Filter and filter holder set after sampling. **e.** Putting the filter holder in HCl 4% for decontamination before using it with another sample. **f.** Sample filter inside plastic zip bag for storage. Different colors of sampled dust might be related to the sources.

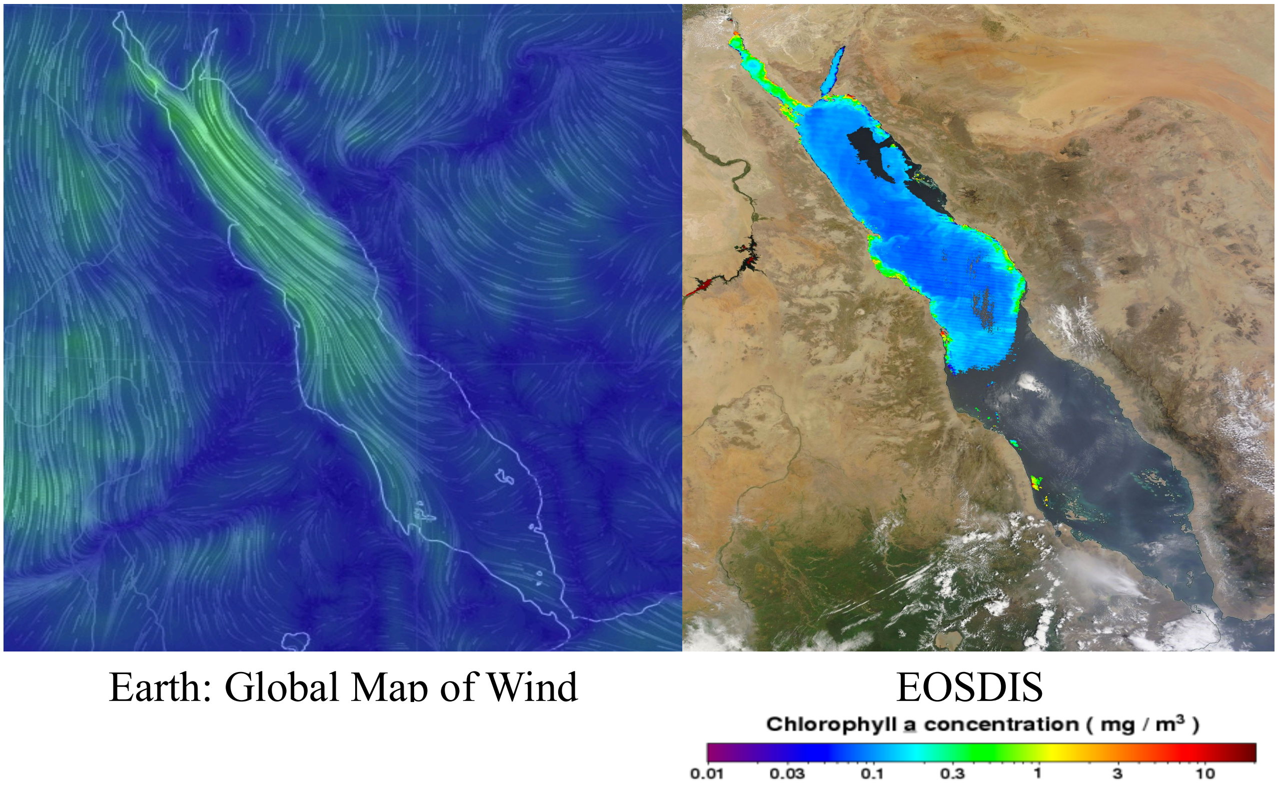

Figure S3. Plankton bloom forming in the northern Red Sea at the offshore sampling during fall 2016. Images are screenshots from earth: a global map of wind, weather, and ocean conditions (https://earth.nullschool.net/#current/wind/surface/level/orthographic=-320.84,24.66,1535/loc=39.240,22.437) and NASA Worldview https://worldview.earthdata.nasa.gov/?p=geographic&l=VIIRS_SNPP_CorrectedReflectance_TrueColor,MODIS_Aqua_CorrectedReflectance_TrueColor(hidden),MODIS_Terra_CorrectedReflectance_TrueColor,MODIS_Aqua_Chlorophyll_A,Reference_Labels(hidden),Reference_Features(hidden),Coastlines(hidden)&t=2016-09-19-T00%3A00%3A00Z&z=3&t1=2016-09-12-T00%3A00%3A00Z&v=24.10381010216834,14.713097150936925,53.12168428259238,29.436346458138786. Maps were generated by zooming into the Red Sea region and selecting the desired date and layer.

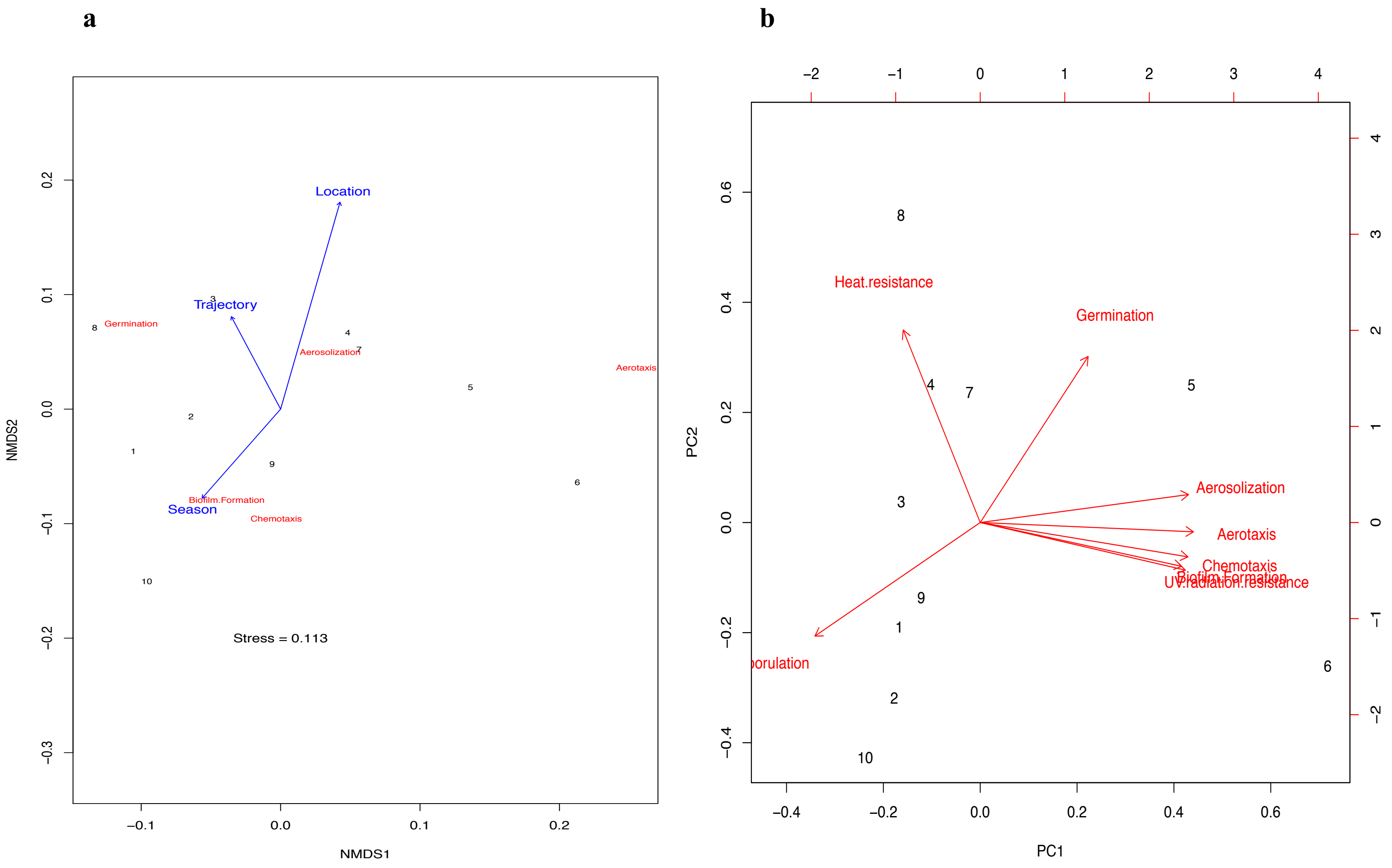

Figure S4. Multi-dimensional scaling . **a**, Non-metric multidimensional scale plot comparing the sampling factors and airborne lifestyle functions. **b**, Principle component analysis of airborne lifestyle functions.
